# Trends in Reported Babesiosis Cases — United States, 2011–2019

**DOI:** 10.15585/mmwr.mm7211a1

**Published:** 2023-03-17

**Authors:** Megan Swanson, Amy Pickrel, John Williamson, Susan Montgomery

**Affiliations:** ^1^Division of Parasitic Diseases and Malaria, Center for Global Health, CDC; ^2^Booz Allen Hamilton, Atlanta, Georgia.

Babesiosis is a tickborne disease caused by intraerythrocytic *Babesia* parasites. In the United States, most babesiosis cases are caused by *Babesia microti*, transmitted from bites of blacklegged ticks, *Ixodes scapularis, *in northeastern and midwestern states. Transmission can also occur through blood transfusions, transplantation of organs from infected donors, or congenital (mother-to-child) transmission (*1*). *Babesia* infection can be asymptomatic or cause mild to severe illness that can be fatal. Overall, U.S. tickborne disease cases have increased 25%, from 40,795 reported in 2011 to 50,856 in 2019 (*2*). Babesiosis trends were assessed in 10 states* where babesiosis was reportable during 2011–2019. Incidence increased significantly in Connecticut, Maine, Massachusetts, New Hampshire, New Jersey, New York, Rhode Island, and Vermont (p<0.001), with the largest increases reported in Vermont (1,602%, from two to 34 cases), Maine (1,422%, from nine to 138), New Hampshire (372%, from 13 to 78), and Connecticut (338%, from 74 to 328). Unlike the other seven states, Maine, New Hampshire, and Vermont, were not included as states with endemic disease in previous CDC babesiosis surveillance summaries. These three states should now be considered to have endemic transmission comparable to that in other high-incidence states; they have consistently identified newly acquired cases every year during 2011–2019 and documented presence of *Babesia microti* in the associated tick vector (*3*). Because incidence in Northeastern states, including Maine, New Hampshire, and Vermont, is increasing, tick prevention messaging, provider education, and awareness of infection risk among travelers to these states should be emphasized.

Babesiosis can cause illness ranging from asymptomatic or mild to severe; the disease can be fatal, particularly among persons who are immunocompromised or asplenic. Common symptoms include fever, muscle and joint pain, and headache. In certain patients, severe complications can occur, including thrombocytopenia, renal failure, and acute respiratory distress syndrome (*1*). Babesiosis can be treated using a combination of antimicrobial medications, such as azithromycin and atovaquone (*2*).

The first case of human babesiosis acquired in the United States was identified in 1969 on Nantucket Island, Massachusetts (*4*). In 2011, babesiosis became a nationally notifiable condition. Where babesiosis is reportable, cases are reported to CDC by state health departments. Until now, CDC considered babesiosis to be endemic in seven states: Connecticut, Massachusetts, Minnesota, New Jersey, New York, Rhode Island, and Wisconsin (*5*). In 2019, the U.S. Food and Drug Administration (FDA) recommended screening blood donations for *Babesia* in states where residents were considered to be at high risk for *Babesia* infection. As a result, FDA recommended blood donation screening in the following 15 states or jurisdictions: Connecticut, Delaware, Maine, Maryland, Massachusetts, Minnesota, New Hampshire, New Jersey, New York, Pennsylvania, Rhode Island, Vermont, Virginia, Wisconsin, and the District of Columbia (*6*).

Previous studies have examined babesiosis transmission and found increasing case counts or rates in particular geographic areas, such as New York (*7*) in previous years (2011–2015) (*4*) and among specific populations, such as those enrolled in Medicare (*8*). The current study identifies trends in babesiosis in the United States during 2011–2019 and highlights establishment of endemic transmission in new geographic areas. Tracking babesiosis transmission over time provides important data to monitor the transmission risk in areas with and without endemic disease.

This analysis used data from the previously described national babesiosis surveillance system (*4*). These data included reported cases from the 41 states where babesiosis was reportable during 2011–2019 (*5*); data reported by the state of New York and New York City were merged and are referred to as New York. Trends were tracked over time by including in the analysis all states that met the following criteria: 1) data were submitted for the entire analytic time span (2011–2019), and 2) 10 or more babesiosis cases were reported for ≥2 consecutive years. Using these criteria, case data reported by Connecticut, Maine, Massachusetts, Minnesota, New Hampshire, New Jersey, New York, Rhode Island, Vermont, and Wisconsin were included. Yearly incidence and overall percent rate change from 2011 to 2019 were calculated for each state. State babesiosis rates were modeled with Poisson regression. An overall model was fit, controlling for state, with year of diagnosis as a continuous variable. State-level models were also fit, controlling for event year (symptom onset or laboratory diagnosis date) as a continuous variable. The natural logarithm of the state’s census population for each year was used in the offset (a variable used when data are recorded over an observed period) to control for state population. All analyses were conducted using SAS (version 9.4, SAS Institute). This activity was reviewed by CDC and was conducted consistent with applicable federal law and CDC policy.^†^

During 2011–2019, a total of 16,456 cases of babesiosis were reported to CDC by 37 states, including 16,174 (98.2%) reported from the 10 states included in this analysis (Figure). New York reported the largest number of cases (4,738 total; average = 526.4 per year), followed by Massachusetts (4,136; 459.6), and Connecticut (2,200; 244.4). The lowest numbers of cases were reported in Vermont (114; 12.7) and New Hampshire (340; 37.8). Incidences ranged from 0.32 per 100,000 population in Vermont in 2011 to 18.0 in Rhode Island in 2015 (Table). The three states with the highest reported incidences were Rhode Island (18.0 per 100,000 population in 2015), Maine (10.3 in 2019), and Massachusetts (9.1 in 2019).

**FIGURE:**
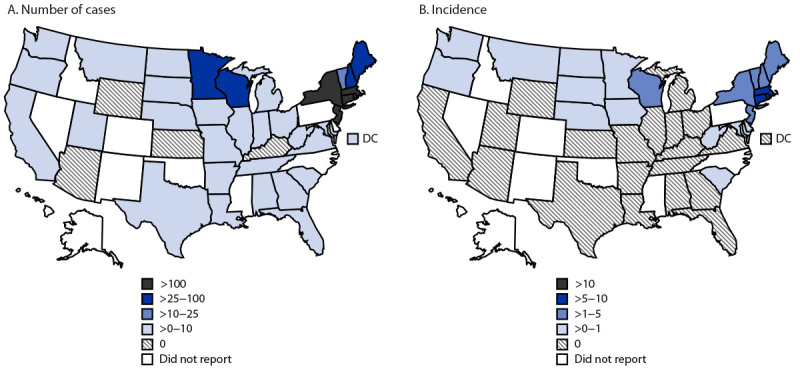
Average number of reported babesiosis cases (A)* and average babesiosis incidence (B),^†^ by state — United States, 2011–2019 **Abbreviation: **DC = District of Columbia. * Cases classified by state of residence (16,456). ^†^ Cases per 100,000 population.

**TABLE:** Reported babesiosis cases and incidence, by year — 10 states,* 2011–2019

State*^,†^	Total no. of cases reported	Average annual case count (range)	Incidence^§^ (no. of cases)									Average annual incidence (range)	Total 9-yr incidence change, %	p-value^¶^
			2011	2012	2013	2014	2015	2016	2017	2018	2019			
Connecticut	**2,200**	244.4 (74–328)	2.1 (74)	3.4 (123)	7.5 (268)	5.7 (205)	9.1 (328)	9.0 (322)	8.6 (309)	6.9 (248)	9.0 (323)	6.8 (2.1–9.1)	**338.4**	<0.001
Maine	**591**	65.7 (9–138)	0.7 (9)	0.8 (10)	2.7 (36)	3.2 (42)	4.1 (55)	6.2 (82)	8.8 (118)	7.5 (101)	10.3 (138)	4.9 (0.7–10.3)	**1,421.6**	<0.001
Massachusetts	**4,136**	459.6 (208–636)	3.1 (208)	3.9 (261)	6.2 (417)	7.9 (535)	6.5 (444)	7.6 (517)	8.6 (591)	7.6 (527)	9.2 (636)	6.7 (3.1–9.2)	**193.0**	<0.001
Minnesota	**486**	54.0 (41–73)	1.4 (73)	0.8 (41)	1.2 (64)	0.9 (49)	0.8 (45)	0.9 (50)	1.1 (60)	0.9 (49)	1.0 (55)	1.0 (0.8–1.4)	**−28.2**	0.176
New Hampshire	**340**	37.8 (13–78)	1.0 (13)	1.4 (19)	1.7 (22)	3.2 (42)	4.0 (53)	1.0 (13)	5.8 (78)	2.7 (37)	4.6 (63)	2.8 (1.0–5.8)	**371.5**	<0.001
New Jersey	**1,719**	191.0 (92–247)	1.9 (166)	1.0 (92)	1.9 (171)	1.8 (159)	3.1 (281)	1.9 (174)	2.1 (193)	2.8 (247)	2.6 (236)	2.1 (1.0–3.1)	**40.9**	<0.001
New York	**4,738**	526.4 (253–696)	2.1 (418)	1.3 (253)	2.7 (534)	2.4 (471)	2.9 (581)	2.2 (430)	3.5 (696)	3.3 (641)	3.4 (663)	2.7 (1.3–3.5)	**58.3**	<0.001
Rhode Island	**1,272**	141.3 (56–190)	6.9 (73)	5.3 (56)	13.5 (142)	16.3 (172)	18.0 (190)	14.7 (155)	15.2 (161)	15.6 (165)	14.9 (158)	13.4 (5.3–18.0)	**115.7**	<0.001
Vermont	**114**	12.7 (2–34)	0.3 (2)	0.3 (2)	1.0 (6)	0.5 (3)	1.4 (9)	2.4 (15)	3.5 (22)	3.4 (21)	5.4 (34)	2.0 (0.3–5.4)	**1,601.8**	<0.001
Wisconsin	**578**	64.2 (43–88)	1.4 (80)	0.8 (45)	1.3 (76)	0.7 (43)	1.0 (56)	1.2 (68)	1.5 (88)	1.1 (64)	1.0 (58)	1.1 (0.7–1.5)	**−28.9**	0.892

* Babesiosis is not a reportable condition by law in the following states: Alaska, Colorado, Hawaii, Idaho, Mississippi, Nevada, New Mexico, North Carolina, Oklahoma, and Pennsylvania.

^†^ The following states or jurisdictions did not meet inclusion criteria for the analysis (cases reported all years during 2011–2019 and ≥10 cases per year for ≥2 years): Alabama, Arizona, Arkansas, California, Delaware, District of Columbia, Florida, Georgia, Illinois, Indiana, Iowa, Kansas, Kentucky, Louisiana, Maryland, Michigan, Missouri, Montana, Nebraska, North Dakota, Ohio, Oregon, South Carolina, South Dakota, Tennessee, Texas, Utah, Virginia, Washington, West Virginia, and Wyoming.

^§^ Cases per 100,000 population.

^¶^ P-values calculated using Poisson regression for each state, controlling for year and state.

Vermont, Maine, and New Hampshire experienced the largest percent change in incidence between 2011 and 2019. Vermont reported two cases in 2011 (incidence = 0.3 per 100,000 population) and 34 cases in 2019 (5.4), representing a 1,602% increase in incidence. Maine reported nine cases in 2011 (0.7) and 138 cases in 2019 (10.3), a 1,422% rate increase. Reported cases in New Hampshire increased from 13 in 2011 (1.0) to 63 in 2019 (4.6), a 372% rate increase. Connecticut, Maine, Massachusetts, New Hampshire, New Jersey, New York, Rhode Island, and Vermont reported significant changes in annual babesiosis incidence. Annual incidence did not change significantly in Minnesota and Wisconsin. Incidence trended upward in Connecticut, Maine, Massachusetts, New Hampshire, New Jersey, New York, Rhode Island, and Vermont, whereas incidence in Minnesota and Wisconsin remained stable.

## Discussion

Monitoring patterns of disease over time is critical to understanding regional changes in infection risk. Clinicians can use knowledge about current infection risk to aid in patient diagnoses, and public health authorities can base prevention activities on risk. Increasing babesiosis case counts and incidences have been documented in other smaller scale studies (*4*,*7*,*8*), but this report is the first comprehensive national surveillance assessment and multistate analysis of babesiosis over time. During 2011–2019, babesiosis incidence significantly increased in states with endemic transmission, as well as in certain neighboring states. Connecticut, Massachusetts, and New York reported the largest numbers of cases as well as significantly increasing incidences. The highest incidences have been reported from Rhode Island (18.0 cases per 100,000 population), Maine (10.3), and Massachusetts (9.2). Reported case counts in Maine, New Hampshire, and Vermont were similar to or higher than those in states previously identified as having endemic babesiosis, and annual incidences in these states have increased significantly.

Because case counts and rates have increased, clinicians need to be aware of the signs and symptoms of and risk factors for babesiosis in their practice areas, particularly as other tickborne conditions can have similar clinical manifestations, risk for disease acquisition, and geographic distribution (*1*). This awareness applies to states bordering those with endemic disease, where increased case counts and infection rates have been documented. Low numbers of cases have been reported from areas where no, or rare, sporadic cases of babesiosis had been reported, including the Canadian provinces of Manitoba and Ontario (*9*) as well as Delaware, Illinois, Iowa, Maryland, North Dakota, Ohio, South Dakota, Virginia, and West Virginia (*5*).

The expansion of babesiosis risk could have implications for the blood supply. *Babesia* is transmissible via blood transfusion, and persons who acquire babesiosis through contaminated blood have been shown to have significantly worse health outcomes and a higher risk for death than do those who acquire the disease from a tick bite (*1*). Currently, the FDA recommends blood donation screening for babesiosis in 14 states and the District of Columbia (*6*). Babesiosis risk in Maine, New Hampshire, and Vermont is comparable to that in the northeastern and midwestern states where babesiosis has been considered endemic, and FDA guidance recommends blood donor screening for *Babesia* infection in those states (*6*). Ongoing evaluation of both tickborne and transfusion transmission risks in states that border those with endemic transmission is important for the evaluation and evolution of babesiosis blood screening policy.

The parasite *B. microti* has been identified in ticks within Maine, New Hampshire, and Vermont (*3*). Based on the increasing numbers of cases, trends in rates, and the parasite’s presence in ticks within the states, CDC now considers babesiosis to be endemic in these states.

The findings in this report are subject to at least three limitations. First, babesiosis is not reportable in all states; for example, although transmission of *B. microti* has been documented in Pennsylvania, babesiosis is not a reportable condition in that state (*6*,*10*). Second, these data probably do not represent all incident cases of babesiosis in reporting states. Patients with nonspecific symptoms might not be tested for babesiosis. Finally, cases are reported by the patient’s state of residence and might not always reflect the location where transmission occurred.

Members of the public and health care providers in states with endemic babesiosis and bordering states should be aware of the clinical signs of babesiosis and risk factors for *Babesia* infection. Persons spending time outdoors in states with endemic babesiosis should practice tick bite prevention, including wearing long pants, avoiding underbrush and long grass, and using tick repellents.

**Summary**

**What is already known about this topic?**

Babesiosis is an emerging zoonotic tickborne parasitic disease in the United States and occurs primarily in the Northeast and Midwest.

**What is added by this report?**

During 2011–2019, U.S. babesiosis incidence significantly increased in northeastern states. Three states (Maine, New Hampshire, and Vermont) that were not considered to have endemic babesiosis had significantly increasing incidences and reported case counts similar to or higher than those in the seven states with known endemic transmission.

**What are the implications for public health practice?**

As case rates rise in multiple states, tick prevention messaging, provider education, and traveler risk awareness should be emphasized.

## Footnotes

* Connecticut, Maine, Massachusetts, Minnesota, New Hampshire, New Jersey, New York, Rhode Island, Vermont, and Wisconsin.

† 45 C.F.R. part 46, 21 C.F.R. part 56; 42 U.S.C. Sect. 241(d); 5 U.S.C. Sect. 552a; 44 U.S.C. Sect. 3501 et seq.
