# *Erratum:* Vol. 72, No. 11

**DOI:** 10.15585/mmwr.mm7215a7

**Published:** 2023-04-14

In the report, “Trends in Reported Babesiosis Cases — United States, 2011–2019,” on page 273, the first sentence in the third paragraph should have read, “The first case of human ***Babesia microti* infection** acquired in the United States was identified in 1969 on Nantucket Island, Massachusetts (*4*).”

